# Enhancing an enterprise data warehouse for research with data extracted using natural language processing

**DOI:** 10.1017/cts.2023.575

**Published:** 2023-06-13

**Authors:** Tanja Magoc, Russell Everson, Christopher A. Harle

**Affiliations:** 1 College of Medicine, University of Florida, Gainesville, FL, USA; 2 UF Health, Gainesville, FL, USA; 3 Richard M Fairbanks School of Public Health, IUPUI, Indianapolis, IN, USA

**Keywords:** Natural language processing, enterprise data warehouse for research, electronic health records, data service, smoking behavior, rule-based, ETL

## Abstract

**Objective:**

This study aims to develop a generalizable architecture for enhancing an enterprise data warehouse for research (EDW4R) with results from a natural language processing (NLP) model, which allows discrete data derived from clinical notes to be made broadly available for research use without need for NLP expertise. The study also quantifies the additional value that information extracted from clinical narratives brings to EDW4R.

**Materials and methods:**

Clinical notes written during one month at an academic health center were used to evaluate the performance of an existing NLP model and to quantify its value added to the structured data. Manual review was utilized for performance analysis. The architecture for enhancing the EDW4R is described in detail to enable reproducibility.

**Results:**

Two weeks were needed to enhance EDW4R with data from 250 million clinical notes. NLP generated 16 and 39% increase in data availability for two variables.

**Discussion:**

Our architecture is highly generalizable to a new NLP model. The positive predictive value obtained by an independent team showed only slightly lower NLP performance than the values reported by the NLP developers. The NLP showed significant value added to data already available in structured format.

**Conclusion:**

Given the value added by data extracted using NLP, it is important to enhance EDW4R with these data to enable research teams without NLP expertise to benefit from value added by NLP models.

## Introduction

Unstructured data are reported to contain about 80% of clinical information stored in electronic health records (EHR) [1]. Natural language processing (NLP) methods have been applied to develop models to extract medical terminology from clinical narratives, such as disease states and medication names, and to describe health conditions or behaviors [2–17]. These models have been successfully used in many research studies to unlock important information hidden in unstructured clinical narratives. However, development of an NLP model is a complex process often bottlenecked by manual annotation of notes for model training [18]. Even when an accurate model exists, generalizability and portability to new populations and information systems environments have been a longstanding challenge [19–20] because documentation practices vary widely across providers and availability of recommended compute infrastructure and trained informatics staff may differ among institutions [20–22]. Even when an NLP model is applied at the same institution where it was trained and developed, it can still be time-, compute-, and labor-intensive to apply NLP models to clinical narratives by a different research team or in a different project [23], which may limit its adoption and use.

Several studies have focused on standardizing information extracted from clinical narratives to internationally adopted common data models, such as Informatics for Integrating Biology and the Bedside (i2b2) [14] and Observational Medical Outcomes Partnership (OMOP) [24], or to local tabular format [25–26]. Many of these studies have shown the improved results obtained in data analysis and in defining a computable phenotype when the model incorporated information extracted from clinical narratives in comparison to using only data available in structured format in EHR. However, each of these studies focuses on a specific disease, and the information extracted from clinical narratives was only used by the research team that developed the NLP model.

Health sciences researchers can benefit when NLP is applied to enterprise-wide clinical data, especially for commonly used social or behavioral concepts. This study presents an architecture for enhancing an enterprise data warehouse for research (EDW4R) [27] with results from an NLP model, which allows discrete data derived from clinical notes to be made broadly available for research use. We focus on the integration of the NLP to extract smoking behavior into the extract, transform, load (ETL) process in the local EDW4R at the University of Florida Health (UF Health). These data are not reliably collected in structured form, however, their availability as structured data allows computation of clinical and research-relevant information such as identifying patients who fit guidelines for lung cancer screening [28–33] and recruitment for cancer-related clinical trials [34–37]. We explain how this architecture can be generalized for future NLP models that extract different concepts. We also quantify the additional value that information extracted from clinical narratives brings to EDW4R by comparing data already available in structured format describing smoking behaviors to the results of the NLP model.

## Materials and methods

### Setting

UF Health is an academic health center, which includes 11 hospitals and hundreds of multispecialty physician practices and outpatient locations in urban and rural areas in North and Central Florida, managing more than three million inpatient and outpatient visits per year and serving patients from all 67 Florida counties and beyond [38–39]. The UF Health EDW4R (also named the Integrated Data Repository) contains more than two billion facts on about 2.4 million patients cared for in the last 12 years. In addition to commonly used structured data, such as demographics, diagnoses, procedures, medications, laboratory results, and vitals, the EDW4R contains unstructured clinical narratives.

The UF Health EDW4R contains more than 250 million unstructured clinical narratives written over more than 12 years, including progress, history and physical, and nursing notes among others. These narratives include content written in all settings, including inpatient, outpatient, and emergency department, and including more than 50 different departments and specialties. All narratives that are classified as “notes” by Epic, the electronic health record (EHR) provider used by UF Health, were included in the study.

The UF Health EDW4R also contains other unstructured data such as procedure narratives, pathology results, and imaging impressions. These narratives, which are not classified as “notes” by Epic, were not currently included in the study. While some of this free-text content might contain relevant information, the NLP model was not trained on these types of documents, so we decided to pilot the process using only data from one Epic table.

### Process to enhance EDW4R with NLP-extracted information

**Figure 1.**
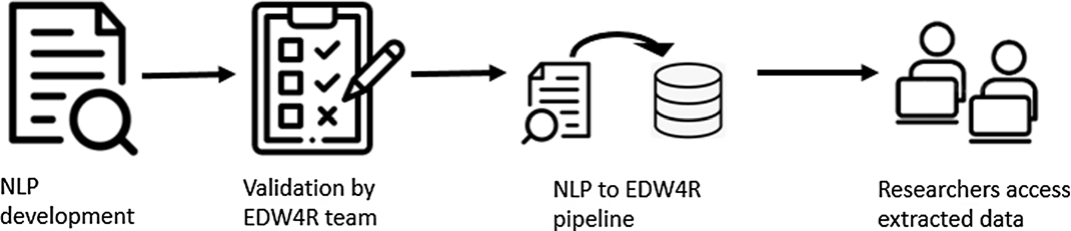
A reliable process to enhance enterprise data warehouse for research (EDW4R) with results of a natural language processing (NLP) model and enable it for use in research.

We developed a process to enhance our institutional EDW4R with the results of an existing NLP model. We present the approach using an NLP code that targets extraction of smoking behavior information from clinical narratives. Figure 1 shows four main steps in the process: NLP model development by the NLP researcher team, independent validation of the model by the EDW4R team, running the pipeline to load NLP extracted data into EDW4R, and enabling the use of NLP extracted information by all researchers.

### NLP model

The NLP model was trained, validated, and disseminated by NLP researchers in collaboration with clinical experts [5]. The NLP package for extracting smoking information is a rule-based NLP model that contains a Java executable file, a set of files containing rules, and a documentation file describing the executable environment requirements, the format of the input and output files, and the instructions on how to run the code. The code requires a .csv file as the input containing five columns: a note identifier, a patient identifier, note date, note type, and note text. The output is a .tsv file containing five columns: a note identifier, a patient identifier, note date, extracted data type, and extracted data value. The extracted data type is one of five data elements on which the NLP model was trained to extract related to smoking habits: smoking years (SYs) are the number of years the person had smoked; packs-per-day (PPD) is the average number of cigarette packs that the person smokes each day; pack-years (PYs) are the product of PPD and SYs; year at quit (YQ) is the year when the person quit smoking; quit year (QY) is how many years ago the person quit smoking. These data elements are often used to identify patients eligible for lung cancer screening and clinical trials.

### NLP model validation by EDW4R team

Our EDW4R team validated the design and performance of the model independently of the validation previously performed by the model developers. We processed all notes written during one month. For each data category (e.g., PPD) and for each note type (e.g., progress note), we counted the number of notes for which the NLP model extracted a value for that category. Next, we randomly selected 1% of each note type and performed manual extraction of values from the category. From the selected 1% of notes for manual annotation, we cleaned the NLP results by eliminating those results that did not fit the expected range of values. For QY, the expected result is a four-digit year, and all other data categories are expected to contain a nonnegative number. However, during initial validation, we noticed that for some categories, the NLP extracted adjectives, such as “approximately” instead of a numeric value. After removing the NLP results that did not comply with the expected data values, we computed positive predictive value (PPV) by utilizing manual annotation as our gold standard. We only focus on PPV since NLP results are extracted to supplement existing structured data.

### Implementation of system to enhance EDW4R with NLP extracted data

**Figure 2.**
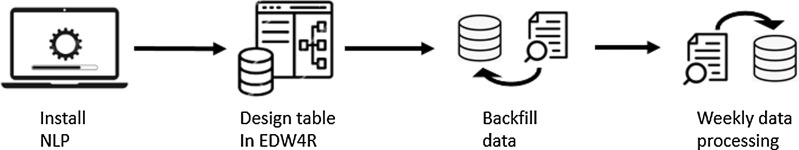
Natural language processing **(**NLP) to enterprise data warehouse for research (EDW4R) system.

Figure 2 shows steps in implementing the system to enhance EDW4R with data extracted by an existing NLP model. After the NLP model is installed in the proper environment and the final database destination is designed, the system runs the NLP model to backfill the database with data from notes already existing in EDW4R and then runs a weekly ETL process to insert information extracted from the new notes into the database.

The NLP model provided by the developers was installed in the EDW4R ETL environment and its features and process of running and interpreting results were learned by our independent EDW4R team in collaboration with the model developers. The installation was straightforward since the executable environment only needs Java and minimal central processing unit and memory requirements. However, we installed the code on a much more powerful graphical processing unit (GPU) server to make this process generalizable to future NLP packages that we might deploy, which might require more computing power or programming libraries that require GPUs.

The database was designed so that the results of NLP can be easily linked to other EHR data already existing in the database. Even though the output of the specific NLP model includes data such as patient identifier, note date, and note type, these data elements were not included in the database. We only included the note identifier, extracted data type, and extracted data value in the database, and we rely on using the note identifier to link the NLP-extracted information to other EHR data such as patient, encounter, and note metadata.

The NLP model was initially run on 255 million clinical narratives that existed in our EDW4R at the time of the initial run, and the resultant data were backfilled. We included inpatient and outpatient clinical narratives, such as progress, history and physical, telephone, consult, discharge, and nursing notes. For the initial implementation, we focused on clinical narratives stored in the EHR’s notes tables and therefore did not include imaging, pathology, and other narratives describing specific procedures and their results. Once all existing notes were processed, a pipeline was set to run weekly on all new notes from the last week.

**Figure 3.**
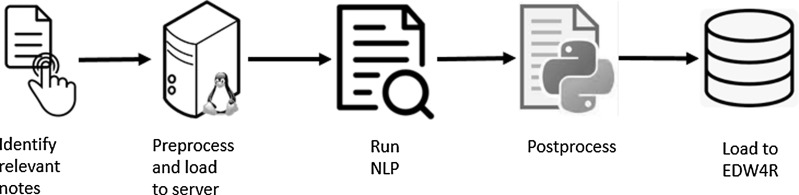
Natural language processing (NLP) data extraction and loading to enterprise data warehouse for research (EDW4R).

Figure 3 shows the steps in processing notes. Both backfill and weekly runs follow the same process. We developed custom Python scripts for all steps in the process except the last step, which is done following an EDW4R-established method using SAP Data Services [40].

The first step in the process is identifying relevant notes. While it was straightforward to use all notes already existing in the database for the backfill stage, for the weekly run, we identify all clinical narratives that were created or might have been updated in the last week using a custom field previously built that denotes when a note was loaded into the EDW4R database. Due to the number of notes and the compute power of the server that runs NLP, we create seven input files with each file containing notes edited during a specific day.

In the next step, preprocessing is performed to generate the file format needed for the NLP model and to load data to the GPU server. Even though the NLP model requires five specific columns as the input, our design of the EDW4R required only two distinct input columns: note identifier and note text. We use dummy values for the other required columns to simplify querying the database to pull all relevant notes data. These steps are completed in parallel on four cores. Finally, files are loaded to the GPU server for NLP processing.

The NLP is launched to process multiple files in parallel. For the weekly run, all seven files are processed simultaneously. Once the NLP is completed on all files, the files are postprocessed to generate the format needed for the EDW4R. The resulting .csv files are available for the SAP data service job to load data into EDW4R. Loading data into EDW4R step also checks the compliance to the primary key constraint and resolves any found issues if possible or throws an error message.

The weekly pipeline is set using a cron job, which is a utility on Linux server that allows a job to be scheduled to run automatically on specific days. The pipeline runs each Monday to identify notes that were written or updated during the past week, and run preprocessing, NLP, and postprocessing steps. Data load to EDW4R is scheduled using an SAP Data Services repository scheduler to run on Tuesdays to ensure proper completion of the previous steps before its start.

### Availability of NLP-extracted data to all researchers

Our team of honest brokers uses a SAP Business Intelligence [41] layer to identify relevant data elements for research teams that we serve. Thus, we have designed a process to expose NLP-extracted data elements within this business layer and link it to other relevant EHR data, including patient information, encounter information, and metadata about the note from which the information was extracted. These data elements can be queried by the team of honest brokers in the same way as all the other structured data elements and therefore served to researchers in the same manner.

### Value added by NLP-extracted information

To assess the value added by the NLP-extracted information, we extracted all structured data describing smoking behavior for all patients seen in our health system over one month in addition to notes that we processed *via* NLP. Structured data that are available in our EDW4R contain only PPD and YQ information. Thus, for further analysis, we focused only on PPD and YQ since these are the only two elements available in both EHR data available as structured data and in NLP extracted data. We kept only data that have nonzero values. We counted the number of unique patients who have smoking behavior information as structured data, as NLP extracted data, and in both methods to calculate the number of patients whose smoking information was a value added to EDW4R *via* NLP pipeline due to smoking information not existing in structured form for these patients. We also compared the values that exist in structured data to those values extracted by the NLP for patients who have smoking information available in both methods to evaluate the agreement between two methods of recording smoking information. We also examined potential reasons for information mismatches between two sources of data.

## Results

### Data summary

**Figure 4.**
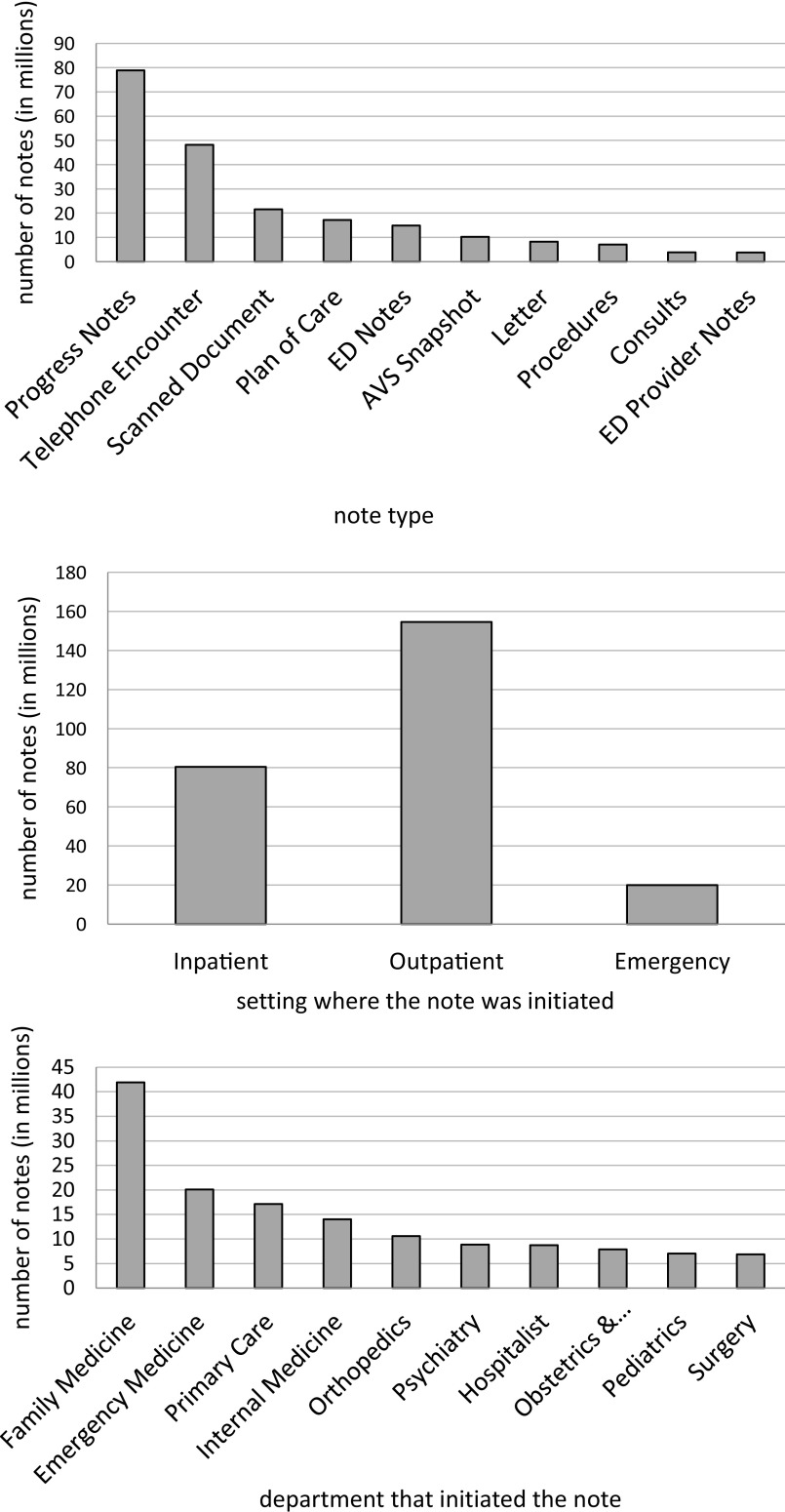
Distribution of notes over three different categories: top 10 note types, settings where the note was initiated, and top 10 departments that initiated the note.

We processed over 255 million clinical narratives during the backfill stage split into about 200 files. The notes accumulated over 600 GB of data and encompassed more than ten years of activity across about two million patients. Figure 4 shows the distribution of notes based on the most common note type, setting in which the note was written, and the leading departments that initiated writing a note.

**Figure 5.**
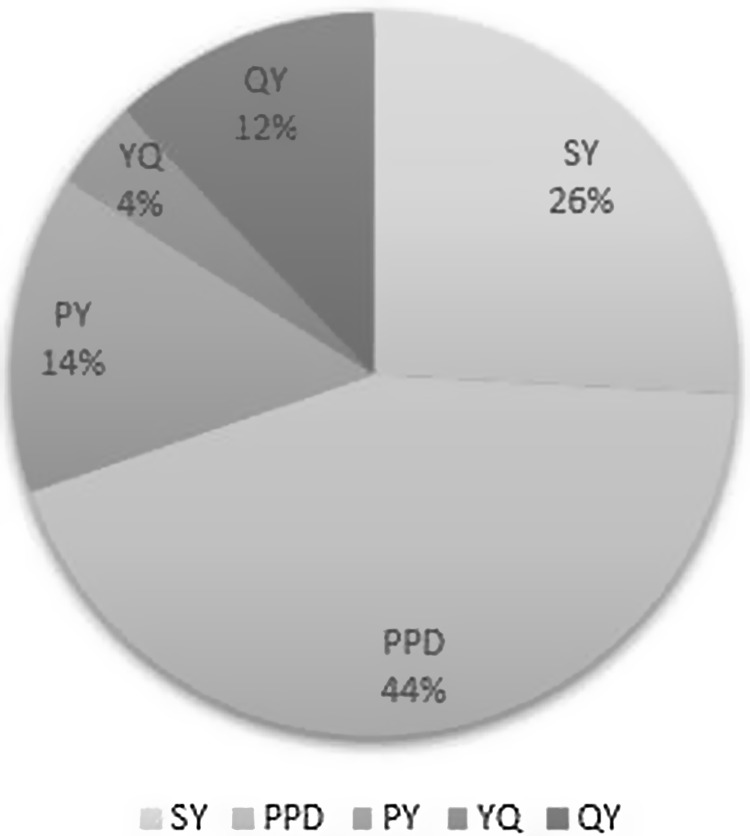
Proportion of extracted data categories from clinical narratives. The categories include: packs-per-day (PPD), pack-years (PY), year at quit (YQ), quit years (QY), and smoking years (SY).

The NLP pipeline resulted in approximately 17.5 million new facts, including SY, PPD, PY, YQ, and QY, that were imported into EDW4R. Figure 5 shows that almost 50% of the extracted values are PPD. The categories with the smallest portion of the extracted data include YQ and QY, which is expected given that many patients are still active smokers.

Since completing the backfill, we completed 38 weekly runs during which we processed on average 8.7 million notes accounting for 20 GB of data and resulting in 90 thousand new facts per week with similar note types, settings, and department distributions.

### Time to process notes

The backfill stage was done manually with preprocessing step completed in about two days on four cores, NLP processing steps done in about nine days on 30 cores, postprocessing step done in one day, and loading data into EDW4R done in three days.

Weekly runs are scheduled to run automatically and take about a half hour for the preprocessing step, 1.5 hours for NLP processing on seven cores, and several minutes for postprocessing and loading data into EDW4R.

### Positive predictive value

We removed 0, 0, 0.4, 43.5, and 0.5% of NLP results from SY, PPD, PY, YQ, and QY, respectively, due to results not matching the expected format. We calculated the PPV for the remaining notes resulting in 96.9, 93.8, 94.8, 80.8, and 98.6% for SY, PPD, PY, YQ, and QY, respectively. Many incorrect extractions by the NLP model were due to multiple values existing in a note for the same data element. In all cases, the NLP model extracted only one of the values provided in the notes. Notably, 100% of incorrect YQ values were the result of this error.

### Comparison of NLP-extracted data to structured data

Table 1 shows the counts of unique patients with nonzero values. The NLP provided data for more than 2000 patients that do not have structured info (10.2% increase) for PPD and more than 550 patients (3.5% increase) for YQ considering one month of data.

**Table 1.** Number of unique patients with smoking information available in Natural Language Processing (NLP), structured data, both sources, and at least one source

	Packs-Per-Day (PPD)	Year at Quit (YQ)
NLP extracted data	16,867	2,103
Structured data	22,524	16,526
Overlap between NLP and structured data	14,564	1,535
NLP or structured data	24,827	17,094

We compared the data values for those patients that have both structured and NLP data available in the given month. Table 2 shows the results of the comparison. PPD has matching value in 97% of cases. For nonmatching cases, 60% of cases are mismatched by no more than a half-pack. YQ has a matching value in 74% of cases with 42% of nonmatching differing only by one year. We manually reviewed ten notes that had a mismatch of more than one year and noted that there were two different quit years listed in the note, but the NLP extracted only one value.

**Table 2.** Comparison between values stored in structured data and extracted from notes using the Natural Language Processing (NLP). Close disagreement is defined as no more than 0.5 packs per day difference for PPD or no more than 1 year difference for YQ

	Packs-Per-Day (PPD)	Year at Quit (YQ)
Agreement	97%	74%
Close disagreement	1.8%	10.9%
Other disagreement	1.2%	15.1%

## Discussion

We created an effective and adaptable process for enhancing our EDW4R by processing clinical narratives with NLP and extracting smoking behavior information. By backfilling data and then routinely running this process, approximately 12 years of smoking behavior information is available to all researchers at our institution. The process obviates the need for researchers interested in smoking behavior for clinical trial recruitment and various observational studies to include NLP experts on their research teams. This process is easily adaptable to NLP models extracting different concepts as well as to different settings at other organizations.

This process also increases data protection by allowing our honest brokers to deliver only discrete data to researchers rather than complete clinical narratives, thus allowing for all data to be completely deidentified. Additionally, the size of delivered data is significantly reduced allowing for quicker data transfer and easier data storage.

We compared PPV obtained during our validation of the pipeline to the values presented in the original validation paper [5], which reported 100, 100, 94, n/a, and 100 percent for SY, PPD, PY, YQ, and QY, respectively. These values are slightly higher than the values we obtained in our analysis, therefore, emphasizing that it is essential to have an independent group to validate the results of an NLP model to avoid potential biases introduced during the training phase such as selection of data, annotation process, and research design [42,43]. By selecting notes from a different time period than the data used for training and validation by the NLP team as well as data for all patients regardless of their medical history as compared to using notes of only lung cancer patients by the NLP team, the EDW4R team reduced the bias potentially introduced by selecting only patients with specific phenotype. During EDW4R validation, notes were annotated by team members who did not interact with the NLP developers, therefore using their own judgment for extracting results from clinical narratives. This approach ensured that the extracted variables follow the most common interpretation of the concepts as defined by end users rather than NLP experts. Lastly, the independent validation ensured that NLP research design did not result in overfitting to data available to the NLP team for training and testing.

We compared the value added by the NLP extraction to the information already available in EHR as structured data. While the NLP extracted five different variables, only two of these variables are available in EHR structured data, thus providing additional information for all patients with smoking information contained in clinical notes. Even though some data that was NLP extracted from notes is already available in structured data, we noted that some patients had smoking-relevant information only in notes, thus significantly increasing the number of patients with smoking behavior information available for research. When looking at the agreement between NLP extracted data and the structured data available in EHR, we noted that most mismatched values differ by less than a half-pack for PPD and by only one year for YQ. These small differences in mismatched values might be contributed to difficulty in estimating how many packs per day a person smokes when the number is less than a full pack and to the difficulty in remembering when exactly the person stopped smoking, therefore, the patient might report different values when asked multiple times for the estimates. This hypothesis is strengthened by manual evaluation of a set of notes that revealed that some notes contain multiple inconsistent values for the variables.

The implemented process is generalizable to other NLP models. In particular, the independent validation method by the EDW4R team can be applied to evaluate performance of any NLP model, and the Python code written for this purpose would only need minor modifications. The pipeline to load NLP-extracted data into EDW4R has many reusable steps and code. The EDW4R design will remain unchanged, and the database already exists in the database. The pipeline would still need to contain backfill and weekly run steps with notes already staged for the backfill stage, therefore simplifying some of the work. Preprocessing and postprocessing steps of the pipeline might have to be slightly modified depending on the input and output format required by a new NLP model, however, since our team of honest brokers provides clinical notes to all researchers in the exact same format, we expect that all NLP models developed at our institution would have same input format requirements. Lastly, loading data to the EDW4R would require a new job in SAP Data Services, but the job would be exactly the same as the existing job with the exception of the input file names. Finally, enabling the use of NLP-extracted information by all researchers would follow the exact process as described.

In this initial implementation, we used only clinical notes from the notes tables in the EHR system. This process is generalizable to other note types such as pathology and radiology result narratives. A majority of the developed Python code would be reusable in its current format or require minor edits to adapt to getting notes text from different sources.

In this study, we loaded discrete data into a locally designed database without standardization to commonly used data models such as i2b2 [44] or Observational Medical Outcome Partnership (OMOP) [45], and we enabled the access *via* the institution’s honest broker process. Future work includes mapping these data elements to standard concepts and enabling researchers to access them with easily accessible self-service tools (e.g., i2b2) as well as mapping to OMOP standard terminologies [46].

The architecture presented in this paper relies on GPU-powered Linux server, SAP Data Suite, and custom Python scripts. These choices were selected to make the use of already existing tools and processes in our team, but they could be replaced with other comparable tools without changing the high-level design of the system and steps in the data processing. However, it is important to consider the ability to run NLP models in a given computational environment. Some novel NLP models use programming approaches that rely on the processing power of GPUs and cannot be executed otherwise, thus high importance should be given to consideration of this part of the system architecture. Other parts of the system are easier to replace with other technologies, which might alter the efficiency of data processing.

Afshar *et al*. [23] examined a very different architecture, using Hadoop network, to evaluate the possibility of implementing enterprise-wide NLP data extraction. Similar to our initial experience, they concluded that processing large data at once could slow down or completely stop data processing, therefore setting up properly the queue or multiple batches of data is highly important. As the authors mention, their approach to apply cTAKES [2] NLP software to extract all medical terminology from notes is susceptible to dictionary updates and would require multiple runs on the same set of notes to re-extract data when new terminology becomes available. In contrast, our approach to extract only one or a set of related medical terms overcomes the problem of re-extracting the already extracted data. However, we would need to process notes multiple times to run different NLP tools that are specialized in extracting specific terms. This approach might result in longer processing time, but it also allows the implementation in sequential order, which might be carried out on smaller architecture. Lastly, while the work of Afshar *et al*. showed that enterprise-wide architecture for NLP processing could be beneficial for downstream research, our work has taken the next step to design the process to automatically process all new notes on the enterprise-wide level.

While this study has several strengths, it also has limitations. First, high PPV and analysis of value added by NLP extracted data indicate benefits of enterprise-wide NLP application. However, we did not evaluate the sensitivity of the NLP model, so we are unsure whether the full benefit of clinical notes analysis is delivered to researchers. Second, even though we were able to identify several systemic reasons for imperfect behavior of the NLP model as measured by PPV, we were unable to overcome these issues. This is an important area of future work. For example, a future study to determine patterns in notes that contain multiple quit years could improve the algorithm by which the NLP model extracts the information from the note.

## Conclusion

We implemented an efficient process for enhancing the EDW4R with discrete data extracted using an NLP model to enable research teams without NLP expertise and large compute environment to benefit from common social and behavioral concepts stored in clinical narratives. We provided a structured description of the design and implementation of each step of the process, including a robust repeatable process for validation of the accuracy performance of the NLP developed by an independent NLP team, routinized extraction and storage of discrete data from clinical notes, and practical method to deliver these results to research teams. We also dove into details of hardware, software, and estimated processing time needs for each step in the process and described how this process can be generalized to different NLP models and note types. This study presented choices and rationale for the process implementation at our institution, and we hope that these details will enable other organizations to adopt this approach to improving access to important clinical concepts hidden in clinical narratives.
